# Construct validity of functional capacity tests in healthy workers

**DOI:** 10.1186/1471-2474-14-180

**Published:** 2013-06-08

**Authors:** Sandra E Lakke, Remko Soer, Jan HB Geertzen, Harriët Wittink, Rob KW Douma, Cees P van der Schans, Michiel F Reneman

**Affiliations:** 1 Research and Innovation Group in Health Care and Nursing, Hanze University Groningen, University of Applied Sciences, P.O. Box 3109, Groningen, 9701 DC, The Netherlands; 2 Department of Rehabilitation Medicine, Center for Rehabilitation, University Medical Center Groningen, University of Groningen, Groningen, The Netherlands; 3 Groningen Spine Center, University Medical Center Groningen, Groningen, The Netherlands; 4 Research Group Lifestyle and Health, University of Applied Sciences, Utrecht, The Netherlands

**Keywords:** Lifting, Physical endurance, Validity, Work capacity evaluation, Work

## Abstract

**Background:**

Functional Capacity (FC) is a multidimensional construct within the activity domain of the International Classification of Functioning, Disability and Health framework (ICF). Functional capacity evaluations (FCEs) are assessments of work-related FC. The extent to which these work-related FC tests are associated to bio-, psycho-, or social factors is unknown. The aims of this study were to test relationships between FC tests and other ICF factors in a sample of healthy workers, and to determine the amount of statistical variance in FC tests that can be explained by these factors.

**Methods:**

A cross sectional study. The sample was comprised of 403 healthy workers who completed material handling FC tests (lifting low, overhead lifting, and carrying) and static work FC tests (overhead working and standing forward bend). The explainable variables were; six muscle strength tests; aerobic capacity test; and questionnaires regarding personal factors (age, gender, body height, body weight, and education), psychological factors (mental health, vitality, and general health perceptions), and social factors (perception of work, physical workloads, sport-, leisure time-, and work-index). A priori construct validity hypotheses were formulated and analyzed by means of correlation coefficients and regression analyses.

**Results:**

Moderate correlations were detected between material handling FC tests and muscle strength, gender, body weight, and body height. As for static work FC tests; overhead working correlated fair with aerobic capacity and handgrip strength, and low with the sport-index and perception of work. For standing forward bend FC test, all hypotheses were rejected. The regression model revealed that 61% to 62% of material handling FC tests were explained by physical factors. Five to 15% of static work FC tests were explained by physical and social factors.

**Conclusions:**

The current study revealed that, in a sample of healthy workers, material handling FC tests were related to physical factors but not to the psychosocial factors measured in this study. The construct of static work FC tests remained largely unexplained.

## Background

**Figure 1:**
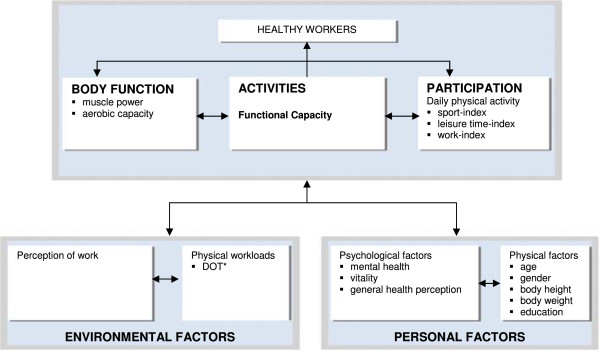
Classification of measures used in this study, according to the International Classification of Functioning, Disability and Health.

Functional Capacity (FC) represents the highest probable level of activity that a person may reach at a given moment in a standardized environment [1,2]. FC is classified within the activity component of the International Classification of Functioning, Disability and Health (ICF) framework [2]. Within ICF, physical activities are influenced by personal factors, environmental factors, body functions, and participation [2] (Figure 1). Thus, FC is considered as a multidimensional construct.

Functional capacity evaluations (FCEs) are assessments of work-related FC such as lifting and static work. Numerous researchers have adopted the ICF and support the consideration of ICF domains when interpreting FC test results [1]. FCEs facilitate the reasoning process for clinicians and assist them in determining if further examination is required [1]. FCEs also assist clinicians in pre-employment screening for healthy workers. In rehabilitation, FCEs assist in selecting diagnoses, recommending ability to work, constructing appropriate treatment plans, and evaluating those treatment plans [3-6].

Several theories and models corroborate the multidimensional construct of work-related FC [7,8]. According to several biopsychosocial viewpoints, optimal work performances are influenced by a worker’s health perception and accomplished in the absence of personal factors such as depression and nervousness [9,10]. The Demand Control Model postulates that environmental factors including ‘a worker’s perception of a heavy workload’ and ‘work-related stress’ need to be at a minimum in order to perform optimally at work [11,12]. Biomechanical models demonstrate relationships between the body functions of muscle power and aerobic capacity with FC test results [3]. Finally, the association of FC tests with participation in daily living activities such as sport, physical work, and leisure time is generally assumed. Until now, the assumed relationships have not been tested in healthy persons. It is of importance to conduct analyzes of the latter assumed relationships in a sample of healthy workers, in order to understand what we are actually testing [13], which is important theoretically to unravel the construct of FC and to develop valid FC tests for healthy workers.

Construct validity is the ability of an instrument to measure a construct [14]. Within the ICF, the FC construct is multidimensional, whereby, one process of FC construct validation is to ascertain how various ICF dimensions may be related to FC test results in healthy workers [14]. From a clinician’s perspective, in healthy workers during pre-employment screening, knowledge of related factors is necessary to identify the necessity of additional testing. From a researcher’s perspective, a comprehensive set of factors related to FC test results in healthy workers may perform as a reference to compare patients´ relationships between FC tests and ICF factors.

The aims of this study were to test relationships between FC tests and other ICF factors in a sample of healthy workers, and to determine the amount of statistical variance in FC tests that can be explained by these factors.

The strength of expected relationships between material handling FC tests (lifting low, overhead lifting, and long carrying) and static work FC tests (standing forward bend and overhead working) with ICF factors are described as hypotheses 1 to 15 in Table 1.

**Table 1:** Hypotheses regarding the strength of relations between functional capacity tests and ICF factors measured in this study

**Hypotheses**	**ICF components**	**Relationships**	**Factor**
H1	Body function	At least fair	1. Muscle power
H2	Body function	At least fair	2. Aerobic capacity
			Daily physical activities
H3	Participation	Low	3. Sport-index
H4	Participation	Low	4. Leisure time-index
H5	Participation	Low	5. Work-index
H6	Environmental factors	Low	6. Perception of work
H7	Environmental factors	Low	7. Physical workloads (DOT)
			Perceived health status
H8	Personal psychological factors	Low	8. Mental health
H9	Personal psychological factors	Low	9. Vitality
H10	Personal psychological factors	Low	10. General health perceptions
H11	Personal physical factors	At least fair	11. Age
H12	Personal physical factors	At least fair	12. Gender
H13	Personal physical factors	At least fair	13. Body height
H14	Personal physical factors	At least fair	14. Body weight
H15	Personal physical factors	Low	15. Education

The value of significant (*P*_*bonf*_ < .002) correlations were interpreted as being low when Pearson, Spearman, or point-biserial correlations between FCEs with ICF factors are ≤ 0.25 and fair when 0.25 < Pearson, Spearman, or point-biserial correlations ≤ 0.50 [14]; DOT, Level of physical workloads according to the Dictionary of Occupational Titles [35]; *H* hypothesis, *ICF* International Classification of Functioning, Disability and Health.

## Methods

### Study sample

During a two-year period, a total of 403 healthy workers (20–60 years of age) executed a 12-item FCE after written informed consent was obtained and the rights of the subjects were protected [15]. We consecutively sampled a series of healthy workers who were employed for at least 20 hours per week and who had taken less than two weeks of sick leave due to musculoskeletal complaints or cardiorespiratory diseases in the year prior to the testing. Prior to the FCE, all workers completed a comprehensive set of questionnaires at home. The Medical Ethical Committee of the University Medical Center Groningen, the Netherlands, approved the research protocol of this study.

### Measures

The variables measured in this study were classified according to the ICF model (Figure 1) [2,16].

### Activities

#### Functional capacity

Functional capacity was measured with five FCE tests, selected to cover a range of physical activities: (1) lifting low; (2) overhead lifting; (3) carrying (material handling tests); (4) standing forward bend; and (5) overhead working (static work tests). These were quantified according to the following:

1) *Lifting low*: Lifting a plastic receptacle from table to floor five times within 90 seconds as the weight is increased in increments 4–5 times.

2) *Overhead lifting*: Lifting a plastic receptacle from table to crown height five times within 90 seconds as its weight is increased in increments 4–5 times.

3) *Carrying*: Carrying a receptacle with two hands for 20 meters as the weight is increased in increments 4–5 times.

4) *Standing forward bend*: For as long as possible, manipulating nuts and bolts while standing, bent forward 30-60° at the trunk, while wearing a five-kilogram weight around the upper thoracic area.

5) *Overhead working*: For as long as possible, manipulating nuts and bolts at crown height while wearing a one-kilogram wrist weight.

A detailed description of the FCE test protocol is published elsewhere [15] and can be requested from the corresponding author. Evaluators (male and female) were third- or fourth-year physical therapy bachelor’s degree students who had received two days of intensive FCE protocol training [15].

The endpoint of testing could be achieved in several manners. First, the subject could express the desire to terminate the activity. Secondly, the evaluator could end the test because the subject’s safety is in jeopardy. Tertiary, 85% of the age-related maximal heart rate was attained. The test-retest reliability of healthy subjects is good for lifting low (ICC = 0.85; 95% CI: 0.89-0.98); overhead lifting (ICC = 0.89; 95% CI; 0.77-0.95); carrying two handed (ICC = 0.84; 95% CI: 0.68-0.93); standing forward bend test (ICC = 0.93; 95% CI: 0.85-0.97); and overhead working (ICC = 0.90; 95% CI: 0.80-0.95) [17,18].

#### Body function

##### Muscle Power

Handgrip strength was measured by the JAMAR hand dynamometer (model PC 5030; Sammons Preston Rolyan, Chicago, IL). Isometric handgrip strength was measured using a protocol where subjects were tested in a seated position with the shoulder adducted and elbow flexed 90°. Forearm and wrist were in the neutral position. In previous studies, the test-retest reliability for handgrip strength (intraclass correlation coefficient [ICC] = 0.97; 95% confidence interval [CI]: 0.94-0.99), intra-, and interrater reliability were good (ICC = 0.85-0.98) in healthy subjects [18,19]. The mean of three measurements of the second grip span of the dominant hand will represent the handgrip strength of the subject [20]. Muscle strength of knee flexion and extension, elbow flexion and extension, and glenohumeral abduction were acquired three times utilizing the Break Method [21,22]. The mean will represent muscle strength. In previous studies, the interrater reliability of the hand-held dynamometer was good for elbow flexion (ICC = 0.95; 95% CI: 0.87-0.98) [23]; elbow extension (ICC = 0.89; 95% CI: 0.74-0.96) [23]; shoulder abduction (ICC = 0.89; 95% CI: 0.74-0.96) [23]; and knee extension (*r*_*p*_ = 0.90) [24]. Elbow measurements were taken with the subject lying in a supine position and elbow flexed 90°, whereby the hand-held dynamometer was situated proximal to the carpus. Knee force was measured with the subject in a sitting position with the knee flexed 90°, whereby the hand-held dynamometer was situated proximal to the calcaneus for flexion and talus for extension. During the shoulder (glenohumeral) abduction test, the shoulder was abducted 90°. The hand-held dynamometer was situated proximal to the lateral epicondyle of the humerus.

##### Aerobic Capacity

In order to estimate maximum oxygen consumption (VO_2max_), a submaximal Bruce Treadmill Test was performed [25]. Beginning at a speed of 2.7 km/h, the speed and slope increased at three-minute intervals until 85% of the estimated age-related maximum heart rate (220 – age) was attained. VO_2max_ was predicted employing the following equation:

$$\begin{array}{ll} \;{\mathrm{VO}}_{2\max}= & 16.62+2.74\;\left(\min\;\mathrm{exercise}\right)–2.584\;\left(\mathrm{men}=1;\mathrm{women}=2\right)–0.043\\ & \left(\mathrm{age}\right)–0.0281\;\\ & \left(\mathrm{body}\;\mathrm{weight}/\mathrm{kg}\right). \end{array}$$

This formula predicted 86% of the VO_2max_ through gasometric measurements [26]. The reproducibility of the prediction equation in healthy men and women is good (*r* = 0.99) [26].

#### Participation

##### Daily Life Physical Activities

In order to measure self-reported physical activity associated with work, sport, and leisure, subjects completed the Dutch language version of the Baecke Physical Activity Questionnaire (BPAQ) [27]. Answers are indicated using a five-point Likert-Scale [27]. The BPAQ consists of three subscales: the work-index, the sport-index, and the leisure-time index. The work-index represents energy expenditure during work and was based on subjects’ workload level, answers to questions regarding working positions, and performance during work. The sport-index was calculated by multiplying the energy expenditure level of the sport with the number of hours per week and proportion of the year in which the sport was played. Higher scores represent greater physical activity [27,28]. The leisure-time index was comprised of four questions (e.g., “During leisure time, I watch television”). The test-retest reliability is good for the work index (ICC = 0.95), the sports index (ICC = 0.93), and the leisure-time index (ICC = 0.98) [29].

#### Environmental factors

##### Perception of Work

The questionnaire of psychosocial workload and work-related stress (VBBA) includes the Dutch Language version of Karasek’s job content questionnaire which is based on the demand control model [9,11,12,30-32]. It consists of 108 questions, each scored on a four-point Likert Scale, measuring six dimensions, including twelve scales and two separate scales of physical effort and job insecurity (Table 2). Each of the scales, with the exception of commitment to the organization (α = .72), has high internal consistency (Cronbach’s alpha ≥ .80.) Unidimensional reliability, analyzed by the Mokken model, is good H(t) ≥ .40 [32,33]. The scales range from 0 to 100, whereby, a score of 100 indicates minimal job variety, decision latitude, social support, job security, job satisfaction, and high psychological and physical workloads or stress.

**Table 2:** **Structure of Dutch questionnaire of perception of work**[32]

**Dimensions**	**Scale**	**Example question**
Psychosocial workloads		
Psychological workloads	Working pace	“Do you have to work fast?”
	Emotional work-load	“Is your work mentally stressful?”
Job variety	Alternation in work	“Do you get to do a variety of different things on jour job?”
	Learning possibilities	“Do you learn new skills in your work?”
Decision latitude	Skill discretion	“Do you have the freedom to decide how to do your job?”
	Decision authority	“Can you make your own decisions concerning your work?”
Social support	Co-worker support	“Can you ask your colleagues for help?”
	Supervisory support	“Can you ask your supervisor for help?”
Work stress		
Stress	Emotional exhaustion	“When I come home they have to give me a break”
	Worrying	“During leisure time, I worry about my work”
Job satisfaction	Job task satisfaction	“Generally, I find it pleasant to start the working day”
	Commitment to organization	“Work at this organisation is very attractive”
Physical load	Physical load	“Do you find your work physically heavy?”
Perception of job insecurity	Job security	“Do you need more job security for the year coming?”

##### Physical Workload

Workers were classified into four levels of physical workload, according to the Dictionary of Occupational Titles (DOT) including sedentary, light, medium, and heavy work [34,35].

#### Personal factors

##### Perceived Health Status

Perceived health status was measured with the Rand 36-item Health Survey (Rand-36) [36-38]. In this study, the scales mental health, vitality, and general health perceptions were included [36-38]. The mental health scale measures feelings of depression and nervousness; the vitality scale measures feelings of energy and tiredness; the general health perception scale assesses an individual’s belief of being healthy. The internal consistency of the mental health, vitality, and general health scales was good (α = 0.81-0.85) in a Dutch population [37,38]. The construct validity is satisfactory [38]. Answers must be given on a five-point Likert scale, varying from “always” to “never.” Each scale was transformed to a range of 0–100 [36]. Higher scores indicated better mental health, vitality, or general health perception.

##### Physical Personal Factors

Age, gender, body height, body weight and level of education data were culminated using questionnaires.

### Statistical analyses

Descriptive statistics were used to describe the population characteristics. We investigated whether each of the questionnaires was affected by floor or ceiling effect by recoding variables (0 = 0; >0 = 1) in cases the median matched the lowest or highest point of a scale. Two authors assessed normality of distributions utilizing histograms [39,40]. Missing data were excluded on a pair-wise basis. Scatter plots between FC test results and ICF factors were created. To answer the research question regarding the relationships between FC test results and other ICF factors, we calculated Pearson (*r*), Spearman (*ρ*), or point-biserial correlation coefficients (*r*_*pbi*_). To avoid Type I errors, we used Bonferroni’s correction [39]. The value of Pearson (*r*), Spearman (*ρ*) and point-biserial correlations(*r*_*pbi*_) were interpreted as being strong for significant (*P*_*bonf*_ < .002) correlations when *r, ρ*, *r*_*pbi*_ > 0.75; moderate when 0.50 < *r, ρ*, *r*_*pbi*_ ≤ 0.75; fair when 0.25 < *r, ρ*, *r*_*pbi*_ *≤* 0.50; and low when *r, ρ*, *r*_*pbi*_ ≤ 0.25 [14]. The values of the correlation coefficients between FC test results and ICF factors, described in hypotheses 1 to 15 will be tested (Table 1). Inter-correlations between ICF factors which were strong (*r, ρ*, *r*_*pbi*_ > 0.75; *P*_*bonf*_ < .002) were determined.

Each of the FC tests were linearly regressed on the Body function, Participation, Environmental and Personal variables by the minimum Bayesian Information Criterion (BIC), which is strongly consistent in finding the best model and often provides interpretable results for practical purposes [41,42]. To evaluate the proportion of variation of FC tests explained, the coefficient of determination (Multiple R-squared) and its variant adjusted for the degrees of freedom, were evaluated for the complete model as well as for the model selected by minimum BIC. The latter provides an impression of the amount of variance explained by the smaller and better interpretable model.

## Results

### Descriptive statistics

A total of 403 workers (209 males and 194 females) were tested. Means, standard deviations, and medians of sample characteristics are depicted in Table 3. All variables were normally distributed, with the exception of co-worker support, supervisory support, worrying, job task satisfaction, and job security. For the latter variables, non-parametric statistics were employed.

**Table 3:** Characteristics of healthy workers (n = 403)

	Total^*^	Male^*^	Female^*^
	**n = 403**	**n = 209**	**n = 194**
*Body function*			
Muscle power			
Handgrip strength (kg)	41.0(12.5)	50.4(9.5)	31.3(6.1)
Knee flexion (N)	226.4(65.3)	261.4(63.0)	189.0(43.4)
Knee extension (N)	311.1(108.1)	360.0(105.4)	258.8(83.8)
Elbow flexion (N)	229.2(57.9)	269.7(46.5)	185.3(30.6)
Elbow extension (N)	157.8(44.1)	185.9(38.0)	127.3(26.7)
Glenohumeral abduction (N)	152.2(45.5)	181.0(37.3)	118.0(26.9)
Aerobic capacity (ml/min/kg)	33.8(7.4)	36.7(7.1)	30.6(6.4)
*Functional capacity*			
Material handling			
Lifting low (kg)	37.5(15.5)	48.1(13.2)	26.2(7.8)
Overhead lifting (kg)	16.3(6.4)	20.7(5.2)	11.6(3.3)
Carrying (kg)	39.6(14.2)	49.2(11.8)	29.3(8.0)
Static work			
Standing forward bend (sec)	374.6(3.4.9)	356.8(273.7)	393.5(334.5)
Overhead working (sec)	247.2(113.1)	269.2(122.4)	223.6(97.0)
*Participation*			
Sport-index^†^	2.9(1.2)	3.0(1.2)	2.8(1.1)
Leisure time-index^†^	3.1(0.6)	3.1(0.7)	3.3(0.6)
Work-index^†^	2.8(0.7)	2.9(0.7)	2.8(0.7)
*Environmental factors*			
*Perception of work*			
Working pace^||^	38.3(12.6)	38.5(12.6)	38.1(12.6)
Emotional work-load^||^	25.8(14.6)	25.5(13.7)	26.2(15.6)
Alternation in work^||^	40.3(19.3)	40.1(19.3)	40.4(19.4)
Learning possibilities^||^	48.3(23.6)	49.5(22.9)	46.9(24.2)
Skill discretion^||^	28.3(27.2)	28.1(27.5)	28.5(27.0)
Decision authority^||^	32.4(26.1)	29.7(27.2)	35.2(24.8)
Co-worker support^||^	0.0(0.0-100.0)^§^	0.0(0.0-100.0)^§^	0.0(0.0-66.7)^§^
Supervisory support^||^	0.0(0.0-87.5)^§^	0.0(0.0-100.0)^§^	0.0(0.0-77.8)^§^
Emotional exhaustion^||^	21.3(25.6)	20.3(25.0)	22.4(26.3)
Worrying^||^	0.0(0.0-100.0)^§^	0.0(0.0-100.0)^§^	0.0(0.0-100.0)^§^
Job task satisfaction^||^	11.1(0.00-100.0)^§^	11.1(0.00-100.0)^§^	11.1(0.0-100.0)^§^
Commitment to organization^||^	33.1(22.8)	31.4(23.4)	34.9(22.0)
Physical load^||^	20.6(19.1)	21.4(19.8)	19.8(18.3)
Job security^||^	0.0(0.0-100.0)^§^	0.0(0.0-100.0)^§^	0.0(0.0-100.0)^§^
Physical workloads (DOT) ^‡^	2(1–4)^§^	2(1–4)^§^	2(1–4)^§^
*Personal factors*			
Mental health^¶^	71.8(9.6)	72.9(8.8)	70.7(10.4)
Vitality^¶^	67.5(12.5)	68.8(12.0)	66.1(12.9)
General health perceptions^¶^	80.0(25.0-100.0)^§^	75.0(35.0-100.0)^§^	80.0(25.0-100.0)^§^
*Physical personal factors*			
Age (years)	41.4(10.6)	42.2(10.8)	40.6(10.3)
Body height (cm)	176.8(9,3)	183.0(6.8)	170.1(6.5)
Body weight (kg)	75.0(13.0)	81.8(11.9)	67.6(9.9)
Education (0–6) ^#^	5.0(1–7)^§^	4(2–7)^§^	5(1–7)^§^

Abbreviations: kg, kilograms; N, Newton; sec, seconds; cm, centimeters.

^*^ Mean (Standard deviation) of variables.

^§^ Median (Range)

^†^ Measured with Baecke Physical Activity Questionnaire (range 0–5) [27].

^||^ Dutch questionnaire of perception of work (VBBA) (range 0–100) [32].

^‡^ DOT Level of physical workloads according to the Dictionary of Occupational Titles [35].

^¶^ Rand-36 (range 0–100) [38].

^#^ Level 1: primary school not completed; level 2: primary school completed; level 3: school for lower general secondary education finished; level 4: intermediate vocation education finished; level 5: higher vocation education finished; level 6: higher education finished.

Table 4 shows correlation coefficients among the five FC variables and all explanatory variables. No strong correlations were discovered within FC and other variables. The following significant and strong inter-correlations between explanatory variables were found: Gender is strongly correlated with handgrip strength *(r*_*pbi*_ = 0.77; *P*_*bonf*_ < .002). Elbow flexion inter-correlated significantly and strong with elbow extension *(r* = 0.78; *P*_*bonf*_ < .002), shoulder abduction *(r* = 0.79; *P*_*bonf*_ < .002), and handgrip strength *(r* = 0.76; *P*_*bonf*_ < .002). Worrying inter-correlated significant and strong with job security *(r* = 0.99; *P*_*bonf*_ < .002).

**Table 4:** Correlations between the variables lifting low, overhead lifting, carrying, standing forward bend, overhead working and ICF variables

		***r, ρ, r***_***pbi***_	**Functional capacity**									
			**Material handling**						**Static work**			
			**Lifting low**		**Overhead lifting**		**Carrying**		**Standing forward bend**		**Overhead working**	
			**Total**	**♂;♀**	**Total**	**♂;♀**	**Total**	**♂;♀**	**Total**	**♂;♀**	**Total**	**♂;♀**
	*Body function*											
H1	Muscle power											
	Handgrip strength (kg)	*r*	0.68**	0.29**;0.32**	0.72**	0.37; 0.35**	0.68**	0.30**;0.32**	−0.03	0.00; 0.02	0.26**	0.10; 0.22**
	Knee flexion (N)	*r*	0.53**	0.25**;0.21**	0.52**	0.22**;0.22**	0.55**	0.26**;0.32**	−0.04	−0.02; 0.01	0.16**	0.06; 0.05
	Knee extension (N)	*r*	0.49**	0.24**;0.27**	0.45**	0.17*; 0.21**	0.48**	0.19**;0.34**	0.03	0.04; 0.09	0.18**	0.11; 0.03
	Elbow flexion (N)	*r*	0.64**	0.26**;0.25**	0.66**	0.28**;0.30**	0.66**	0.29**;0.34**	−0.03	0.05; 0.00	0.15**	0.02; -0.01
	Elbow extension (N)	*r*	0.64**	0.37**;0.20**	0.66**	0.38**;0.26**	0.63**	0.35**;0.21**	−0.07	−0.04; -0.02	0.14**	0.01; -0.00
	Glenohumeral abduction (N)	*r*	0.66**	0.31**;0.24	0.66**	0.38**;0.22*	0.70**	0.40**;0.34**	0.04	−0.09; 0.07	0.22**	0.10; -0.03
H2	Aerobic capacity (ml/min/kg)	*r*	0.42**	0.21**;0.23**	0.40**	0.17*; 0.19*	0.43**	0.21**;0.23**	0.13**	0.10; 0.23**	0.28**	0.16; 0.03**
	*Participation*											
H3	Sport-index^†^	*r*	0.17**	0.18*; 0.20**	0.14**	0.11; 0.18*	0.16**	0.13; 0.23**	0.11*	0.07; 0.16	0.19**	0.14; 0.24**
H4	Leisure time-index^†^	*r*	0.11*	0.03; 0.06	−0.12*	0.04; -0.00	−0.08	0.10; 0.05	0.04	−0.04; 0.09	0.09	0.14; 0.12
H5	Work-index^†^	*r*	0.13*	0.10; 0.13	0.15**	0.14*; 0.12	0.13**	0.11; 0.12	0.07	0.09; 0.07	−0.02	−0.06; 0.00
	*Environmental factors*											
H6	*Perception of work*											
	Working pace^||^	*r*	0.01	−0.06; 0.01	0.00	−0.02; -0.01	−0.07	−0.17; -0.03	0.09	0.09; 0.09	−0.00	0.03; -0.06
	Emotional work-load^||^	*r*	0.01	−0.08; 0.22**	−0.00	−0.09;0.18	−0.00	−0.09; 0.16	0.12*	0.01; 0.20**	0.10*	0.23; 0.22**
	Alternation in work^||^	*r*	−0.06	−0.03; -0.19**	−0.02	0.00; -0.07	−0.06	−0.02; -0.18	−0.12*	−0.06; -0.18	−0.17**	−0.12; -0.25**
	Learning possibilities^||^	*r*	0.01	−0.05; -0.04	0.04	0.02; -0.02	0.03	−0.01; -0.03	−0.10*	−0.14; -0.07	−0.14**	−0.09; -0.24**
	Skill discretion^||^	*r*	0.00	0.02; -0.00	−0.03	0.01; -0.12	−0.01	0.02; -0.06	−0.07	−0.10; -0.04	−0.20**	−0.22**;-0.18
	Decision authority^||^	*r*	0.00	0.01; -0.0	−0.05	0.03; 0.06	−0.06	0.03; 0.03	−0.07	−0.09; -0.07	−0.16**	−0.19**;-0.08
	Co-worker support^||^	*ρ*	−0.03	−0.02; -0.05	0.01	0.00; -0.03	−0.05	−0.16; -0.02	0.00	0.08; -0.07	−0.08	−0.01; 0.02
	Supervisory support^||^	*ρ*	0.02	−0.03; 0.09	0.04	−0.01; 0.05	0.05	0.00; 0.08	−0.01	−0.06; 0.05	−0.07	−0.13; -0.01
	Emotional exhaustion^||^	*r*	−0.05	−0.07; 0.05	−0.04	−0.06; 0.07	−0.07	−0.13; 0.05	0.13*	0.08; 0.17	−0.01	−0.06; 0.08
	Worrying^||^	*ρ*	0.03	0.02; 0.04	0.04	0.04;0.05	0.02	−0.03; 0.05	0.07	0.08; 0.06	0.03	−0.01;0.07
	Job task satisfaction^||^	*r*	0.05	0.03; 0.02	0.05	0.05; -0.02	0.04	0.00; 0.03	−0.08	−0.05; -0.08	−0.11*	−0.10; -0.16
	Commitment to organization^||^	*r*	−0.08	−0.02; -0.07	−0.07	0.01; -0.08	−0.05	0.04; -0.06	−0.02	−0.04; -0.01	−0.03	−0.00; -0.04
	Physical load^||^	*r*	0.08	0.08; 0.03	0.09	0.12; 0.04	0.07	0.07; 0.02	0.04	0.03; 0.05	0.00	−0.03; 0.03
	Job security^||^	*ρ*	0.05	0.08; -0.06	0.03	−0.02; -0.07	0.00	−0.05; -0.07	−0.08	0.00; -0.18	−0.08	0.02; -0.17
H7	Physical workloads (DOT) ^‡^	*ρ*	0.19**	0.13; 0.10	0.21**	0.16; 0.13	0.20**	0.14; 0.10	0.07	0.10; 0.07	0.03	−0.03; 0.02
	*Personal factors*											
H8	Mental health^¶^	*r*	0.06	−0.01; -0.07	0.10	0.07; -0.05	0.10	0.06; -0.03	0.00	−0.01; 0.02	−0.03	−0.04; -0.07
H9	Vitality^¶^	*r*	0.06	0.01; -0.08	0.10	0.09; -0.07	0.08	0.05; -0.04	−0.06	−0.06; -0.05	0.03	0.03; -0.02
H10	General health perceptions^¶^	*ρ*	−0.02	0.11; -0.05	0.01	0.18**;-0.01	0.01	0.05; -0.05	0.01	0.09; -0.03	0.05	−0.12; 0.05
	*Physical personal factors*											
H11	Age (years)	*r*	0.05	−0.16*;-0.13	−0.01	−0.12; -0.06	−0.07	−0.23**;-0.11	−0.06	−0.13; 0.02	0.00	−0.02; -0.01
H12	Gender	*r*_*pbi*_	0.71**		0.72**		0.71**		−0.06		0.20**	
H13	Body height (cm)	*r*	0.62**	0.24**;0.30**	0.58**	0.12; 0.20**	0.61*	0.23**;0.26**	−0.02	−0.08; -0.01	0.15**	−0.02; 0.04
H14	Body weight (kg)	*r*	0.53**	0.27**;0.22**	0.52**	0.23**;0.19**	0.49**	0.18**;0.18	−0.16**	−0.14; -0.17	−0.01	−0.12; -0.20**
H15	Education (0–6)^#^	*ρ*	−0.07	−0.15; 0.14	−0.06	−0.13; 0.16	−0.03	−0.09; 0.22	0.10	0.00; 0.18	0.12	0.14; 0.15

Abbreviations: *r* Pearson’s correlation coefficient, *ρ* Spearman rho, *r*_*pbi*_*,* Point-biserial correlation coefficient.

* Correlation is significant at the *P* < .05 level (2-tailed).

** Correlation is significant at the *P*_*bonf*_ < .002 level (2-tailed).

^†^ Measured with Baecke Physical Activity Questionnaire [27].

^||^ Dutch questionnaire of perception of work (VBBA) [32].

^‡^ DOT Level of physical workloads according to the Dictionary of Occupational Titles [35].

^¶^ Rand-36 [38].

^#^ Level 1: primary school not completed; level 2: primary school completed; level 3: school for lower general secondary education finished; level 4: intermediate vocation education finished; level 5: higher vocation education finished; level 6: higher education finished.

#### Hypotheses tested

##### Material Handling FC tests

Moderate and fair correlations were found between material handling tests regarding gender, body weight, body height, muscle power, and aerobic capacity (Table 4). Low correlations were determined between all three material handling FC tests and the sport-index, similar to physical workloads. Furthermore, low correlations were encountered between the work-index with overhead lifting and carrying. No significant correlations were found between material handling FC tests and all other participating, environmental, and psychological personal factors. Hypotheses 1, 2, 3, 5, 7 and 12 to 14 were not rejected (Table 1). The remaining hypotheses 4, 6, 8 to 11 and 15 were rejected.

##### Static Work FC tests

Fair correlations were ascertained between overhead working with aerobic capacity and handgrip strength. The sport-index and four scales of the perception of work correlated low to overhead lifting. For standing forward bend, all hypotheses were rejected. For overhead working, hypotheses 1 to 3 and 6 were not rejected (Table 1). Hypotheses 4, 5 and 7 to 15 were rejected.

### Regression analyses

Job security, worrying, co-worker, and supervisory support were recoded as dichotomous variables. The results of the multivariate regression analysis are demonstrated in Table 5.

**Table 5:** Regression analyses of ICF-factors on material handling and static work functional capacity

		***B value***	***SE***	***t***	***P value***
**Material handling**					
**Lifting low**	Constant	−58.88	12.74	−4.62	<.001
***R***^***2***^ **= 0.62**	Gender (Male)	8.58	1.62	5.30	<.001
	Body height (cm)	0.26	0.08	3.21	0.001
	Body weight (kg)	0.14	0.05	2.65	0.008
	Glenohumeral abduction strength (N)	0.05	0.02	2.60	0.01
	Elbow extension strength (N)	0.07	0.02	4.61	<.001
	Aerobic capacity (ml/min/kg)	0.28	0.08	3.47	0.001
	Sport-index^†^	1.21	0.45	2.68	0.008
	Physical workloads (DOT) ^‡^	1.72	0.58	2.97	0.003
**Overhead lifting**	Constant	−1.93	1.40	−1.37	0.17
***R***^***2***^ **= 0.62**	Gender (Male)	3.95	0.65	6.09	<.001
	Handgrip strength (kg)	0.13	0.03	4.99	<.001
	Elbow extension strength (N)	0.04	0.01	5.91	<.001
	Aerobic capacity (ml/min/kg)	0.10	0.03	3.46	0.001
	Physical workloads (DOT) ^‡^	0.79	0.23	3.44	0.001
**Carrying**	Constant	−48.56	11.69	−4.15	<.001
***R***^***2***^ **= 0.61**	Gender (Male)	6.09	1.6	3.81	<.001
	Body height (cm)	0.26	0.07	3.80	<.001
	Handgrip strength (kg)	0.17	0.06	2.78	0.006
	Glenohumeral abduction strength (N)	0.06	0.02	3.37	0.001
	Elbow extension strength (N)	0.07	0.02	4.46	<.001
	Aerobic capacity (ml/min/kg)	0.27	0.068	4.00	<.001
	Physical workloads (DOT) ^‡^	1.53	0.52	2.92	0.004
**Standing forward bend**	Constant	439.36	109.63	4.01	<.001
***R***^***2***^ **= 0.05**	Body weight (kg)	−3.86	1.13	−3.41	0.001
	Aerobic capacity (ml/min/kg)	5.66	2.04	2.78	0.006
	Emotional exhaustion^||^	1.57	0.58	2.73	0.007
**Overhead working**	Constant	177.01	39.54	4.48	<.001
***R***^***2***^ **= 0.15**	Body weight (kg)	−1.52	0.49	−3.09	0.002
	Handgrip strength (kg)	2.65	0.56	4.74	<.001
	Aerobic capacity (ml/min/kg)	2.88	0.77	3.74	<.001
	Skill discretion^||^	−0.77	0.19	−4.04	<.001

*R*^*2*^, adjusted R square; *B value*, unstandardized regression coefficient; *SE* Standard error; *P value,* empirical significant level; constant, outcome of the FC tests with all other factors being zero; ^†^ Measured with Baecke Physical Activity Questionnaire; ^‡^ DOT Level of physical workloads according to the Dictionary of Occupational Titles [35]; ^||^ Subscale of the Dutch questionnaire of perception of work (VBBA) [32].

#### Material Handling

The regression models explained 61% to 62% of the variance in the material handling FC test results. In material handling tasks, the explanatory variables were physical factors: gender, body height, body weight, muscle strength, aerobic capacity, sport-index, and physical workloads.

The regression model for lifting low FC test can be interpreted as follows. On average (Table 5), 1 cm taller increases lifting low by 0.26 kg; 1 kg heavier increases lifting low by 0.14 kg; 1 kg (10 N) more shoulder abduction muscle strength increases lifting low by 0.5 kg and 1 kg (10 N) elbow extension muscle strength increases lifting low by 0.7 kg, 1 ml/min/kg more aerobic capacity increases lifting low by 0.28 kg; 1 point higher on the sport-index associates with 1.21 kg more lifting capacity; and 1 point heavier physical workloads increases lifting low by 1.72 kg.

#### Static Work

The regression model explained 5% to 15% of the variance in the static work FC test results. In static work tasks, the explanatory variables were body weight, aerobic capacity, handgrip strength, emotional exhaustion, and skill discretion (Table 5).

The regression model for standing forward bend FC test can be interpreted as, on average (Table 5), 1 kg less body weight increases standing forward bend by 3.86 seconds; 1 ml/min/kg more aerobic capacity increases standing forward bend by 5.66 seconds; 1 point higher on the emotional exhaustion scale (range 0–100) increases standing forward bend by 1.57 seconds.

## Discussion

The aim of this study was to determine the construct validity of FC tests by gaining insight into related ICF factors in healthy workers [1]. In this study, performed with a healthy population, physical factors influenced FC tests more than the measured psychological or social factors. For material handling, the physically modifiable factors of muscle strength, aerobic capacity, sport-index, work-index, and body weight were significantly associated with material handling tasks, as were the non-modifiable factors of gender and body height. The variance of material handling test results in healthy workers was largely explained by physical factors only. It may be noted that the models found by minimum BIC are best but do not exclude models explaining little less variance e.g. muscle strength is replaced by another, based on strong inter-correlations. The variance of static work FC test results was only minimally explained by physical factors and perception of work.

This is the first study into the construct validity of work-related FC tests in a sample of healthy persons. Patients’ relationships between FC test results and ICF factors differ from healthy workers. In a sample of patients with chronic pain depression was, contrary to current results, significant but low correlated to material handling FC tests [43-45]. The latter studies utilized measurements of depression that were strongly related to the mental health scale of the RAND-36 of this study (r = 0.81) [27,36,46]. However, an explanation for finding no associations between FC tests and mental health scale in our study might be, beside the absence of chronic pain, that the small variance encountered of the mental health scale may explain the current results (Table 3). In patients with chronic pain, similar to the results in this study, there is also high evidence that gender correlates with overhead lifting [10,43,47-49]. In our healthy sample, age did not contribute to the explanatory models of FC tests. However, previous studies have described an average decline of 20% in physical work capacity between the ages of 40 and 60 years [50,51]. In healthy populations, material-handling tasks can be regarded as tests of muscle strength, which is, in part, genetically determined [3,52,53]. Similarly, we observed that male subjects lifted 4.9 kg to 10.3 kg more weight than female subjects in all lifting tasks. The functional interdependence of oxygen transport and muscle activity could be indicative of the relationship between aerobic capacity and lifting tests discovered in our study as lifting tests are known to place an increased demand on the aerobic system [54]. As for muscle strength, to the best of our knowledge, no study has yet been conducted into the relationship between muscle strength and FC test results in patients with chronic pain. It is recommended to do so in future studies in a sample of patients with chronic pain.

The theoretical construct of work-related FC tests was built upon assumed relations between FC test results and other ICF dimensions. These relations were based on the ICF model [2], researchers’ consensus [1], and the demand control model [11,55]. Other bio-psychosocial factors than those measured in this study could possibly be related to FC test results. For example, in patients with chronic pain, there was high evidence that self efficacy relates to FC tests, but a study of self efficacy in healthy workers is nonexistent [7]. For social factors, literature is available that substantiates the influence of the therapeutic alliance and evaluator’s fear of injury beliefs on the self-rated activity level of patients, however, a study with objective measurements in a healthy population is missing [56-58]. Furthermore, in regard to personal factors, in patients with chronic low back pain, fear of movement/(re)injury correlated low with static lifting [7,59-62], but the Tampa Scale of Kinesiophobia (TSK) was not measured in current study. Finally, in regard to the domain body functions, muscle endurance was not measured in this study and may correlate with static work FC tests, especially low back muscle endurance [63].

### Limitations

The cross sectional design is not suitable for prediction of future work performance or future work disability. Therefore no conclusions to bio-psychosocial factors that may possibly be influencing future work performance or work disability can be made based on this cross section study. Although the evaluators were well instructed in the test protocol, the results of this study may differ from a sample that was evaluated by experienced evaluators. The last limitation is that other FC tests might give other results.

A particular strength of this present study is the size of the study population (n = 403) and the existence of factors from each component of the ICF. In this study, psychological factors were defined according to the background of an individual’s life and living, and therefore, were indicated as personal factors within the ICF framework and not as an impairment in mental function [1,2]. Physical activity such as sport activity was classified as a participation component. Had we classified these variables differently, however, the study results would not vary.

### Recommendations

We recommend researchers to replicate this study in a different sample of healthy workers to analyze the robustness of current observations. Further study into the effect of training muscle strength and aerobic capacity on work-related FC tests in healthy workers is also recommended. The empirical evidence of the current study supports fair correlations of FC tests with aerobic capacity. By contrast, in patients with chronic pain, aerobic capacity does not correlate with FC [45]. The transition from healthy workers into patients and the change in the amount of association between aerobic capacity and FC test results and pain might be interesting for the prognosis of developing chronic pain. Therefore, we recommend measuring aerobic capacity and FC tests in a cohort study of healthy workers. Based on the results of this study, we recommend that clinicians, during pre-employment screening in healthy persons, test muscle strength and aerobic capacity if a worker scores lower on a material handling and static work FC test than the reference values. Results of this study imply no direct recommendations for clinicians working with patients, but indirectly, the results may be useful to clinicians to be aware that the operationalization of the FC construct in healthy workers differs from patients.

## Conclusions

In healthy workers, it appears that the construct of material handling FC tests is comprised of the physical factors of muscle strength, aerobic capacity, gender, body height, body weight, sport and physical workloads, but, is not comprised of the psychosocial factors included in this study. The construct of static work FC tests remains largely unexplained. Because of the cross sectional design and the healthy study sample in this study, the results should not be interpreted as predictors for future work performance, nor should they be generalized to patients.

## Abbreviations

FC: Functional capacity; ICF: The international classification of functioning, disability and health framework; FCE: Functional capacity evaluations; ICC: Intraclass correlation coefficient; BPAQ: Baecke Physical Activity Questionnaire; DOT: Dictionary of occupational titles; r: Pearson’s correlation coefficient; ρ: Spearman rho; rpbi: Point-biserial correlation coefficient; BIC: Bayesian information criterion; TSK: Tampa Scale of Kinesiophobia; R2: Adjusted R square; B value: Unstandardized regression coefficient; SE: Standard error; P value: Empirical significant level.

## Competing interests

The authors declare that they have no competing interests.

## Authors’ contributions

RS has made substantial contributions to conception and design, acquisition of data and analysis and interpretation of the data, drafting the manuscript and critically revising it with important intellectual content. JHB participated in the design of the study, drafting the manuscript and critically revising it with important intellectual content. HW drafted the manuscript and critically revised it for important intellectual content. RD acquired data, drafted the manuscript and critically revised it with important intellectual content. CvdS drafted the manuscript and critically revised it with important intellectual content. MR has made substantial contributions to conception and design, acquisition of data and analysis and interpretation of the data, drafting the manuscript and critically revised it with important intellectual content. All authors read and approved the final manuscript.

## Pre-publication history

The pre-publication history for this paper can be accessed here:

http://www.biomedcentral.com/1471-2474/14/180/prepub
